# Supplementary material for: An open-source framework for large-scale, flexible evaluation of biomedical text mining systems
Source: J Biomed Discov Collab. 2008 Jan 29;3:1. doi: 10.1186/1747-5333-3-1 (PMC2276192; doi:10.1186/1747-5333-3-1)
Supplement: Additional file 1 — Evaluation platform source code. The additional file contains the software evaluation framework discussed in this paper. The most current release can be downloaded from SourceForge.net [1]. See the accompanying README file for instructions on installation and use. Also included in the distribution is a collection of UIMA wrappers for some commonly used BioNLP tools and annotated corpora. [file 1747-5333-3-1-S1.zip › bionlp_uima_repository-1.0.1/docs/api/src-html/edu/uchsc/ccp/util/nlp/annotation/printer/AnnotationToFileOutput.html]

```
001    package edu.uchsc.ccp.util.nlp.annotation.printer;
002    
003    import java.io.PrintStream;
004    import java.util.List;
005    
006    import edu.uchsc.ccp.util.nlp.annotation.Annotator;
007    import edu.uchsc.ccp.util.nlp.annotation.Span;
008    import edu.uchsc.ccp.util.nlp.annotation.TextAnnotation;
009    import edu.uchsc.ccp.util.nlp.document.GenericDocument;
010    import edu.uchsc.ccp.util.nlp.mention.ClassMention;
011    
012    /**
013     * This Annotation Printer outputs annotation in a format that can be loaded by the AnnotationFromFileLoader. Currently, this will only work for
014     * simple annotations. Use of the XCas is probably the best way to accomplish this task, but this will be used as we are only interested in storing
015     * simple annotations (i.e. not slots) for the moment.<br>
016     * <br>
017     * The output consists of a single annotation per line taking the format:<br>
018     * <br>
019     * documentID|annotatorID|spanStart spanEnd|classMentionName|coveredText
020     */
021    public class AnnotationToFileOutput implements AnnotationPrinterUtil {
022    
023        public void printDocumentCollection(List<GenericDocument> documents, PrintStream ps) {
024            for (GenericDocument gd : documents) {
025                printDocument(gd, ps);
026            }
027        }
028    
029        public void printDocumentCollection(List<GenericDocument> documents) {
030            printDocumentCollection(documents, System.out);
031        }
032    
033        public void printDocument(GenericDocument gd) {
034            printDocument(gd, System.out);
035        }
036    
037        public void printDocument(GenericDocument gd, PrintStream ps) {
038            List<TextAnnotation> annotations = gd.getAnnotations();
039    
040            for (TextAnnotation ta : annotations) {
041                StringBuffer outputStr = new StringBuffer();
042    
043                ClassMention cm = ta.getClassMention();
044                Annotator annotator = ta.getAnnotator();
045                outputStr.append(ta.getDocumentID() + "|" + annotator.getAnnotatorID() + "|");
046    
047                List<Span> spanList = ta.getSpans();
048                if (spanList.size() > 1) {
049                    int earliestSpanStart = Integer.MAX_VALUE;
050                    int latestSpanEnd = -1;
051                    for (Span span : spanList) {
052                        if (span.getSpanStart() < earliestSpanStart) {
053                            earliestSpanStart = span.getSpanStart();
054                        }
055                        if (span.getSpanEnd() > latestSpanEnd) {
056                            latestSpanEnd = span.getSpanEnd();
057                        }
058                    }
059                    outputStr.append(earliestSpanStart + " " + latestSpanEnd + "|");
060                } else {
061                    outputStr.append(ta.getAnnotationSpanStart() + " " + ta.getAnnotationSpanEnd() + "|");
062                }
063    
064                outputStr.append(cm.getMentionName() + "|" + ta.getCoveredText());
065    
066                ps.println(outputStr.toString());
067    
068            }
069        }
070    
071    }
```
